# Utilising bryophyte herbarium material as a source of fungal novelty: a case study presenting new records of *Bryobroma gymnomitrii* (Döbbeler) Döbbeler on *Gymnomitrion* Corda in Britain and North America

**DOI:** 10.1080/03736687.2024.2375407

**Published:** 2024-07-29

**Authors:** George R. L. Greiff, W. Cuyler Bleecker, Michael Haldeman, Katherine Slade, Nathan Smith, Stefanie M. Ickert-Bond

**Affiliations:** a University of Bristol, 24 Tyndall Avenue, Bristol BS8 1TQ, England, UK; b Herbarium (ALA), University of Alaska Museum of the North, 1962 Yukon Drive, Fairbanks, AK 99775, USA; c University of Alaska Fairbanks, 1962 Yukon Drive, Fairbanks, AK 99775, USA; d Oregon State University, Department of Botany and Plant Pathology, 4617 Cordley Hall, Corvallis, OR 97331-2902, USA; e Amgueddfa Cymru – Museum Wales, Natural Sciences Department, National Museum Cardiff, Cathays Park, Cardiff CF10 3NP, Wales, UK

**Keywords:** Ascomycete, bryoparasitic, bryophilous, herbaria, liverwort

## Abstract

**Introduction:**

Bryophyte herbarium material often contains inadvertently collected bryophilous fungi preserved with their host plant tissues, providing snapshots of the biotic aspects of bryophyte ecology in space and time. During an ongoing project to study bryophilous fungi on leafy liverworts in the genus *Gymnomitrion*, new records of the bryophilous ascomycete *Bryobroma gymnomitrii* (≡ *Bryomyces gymnomitrii*) were discovered in herbarium material from areas in which the fungus was previously unknown. While many host vouchers were screened, the fungus was observed in only five collections. Using *B. gymnomitrii* as a case study, some of the advantages and limitations associated with the utility of bryophyte collections for bryo-mycological analyses are considered.

**Methods:**

More than 400 herbarium specimens from across Europe and North America were screened for the presence of fungi by different researchers at the following four herbaria: ALA, BBSUK, NMW and OSC.

**Key results and conclusions:**

*Bryobroma gymnomitrii* appears to be specific to *Gymnomitrion concinnatum* and *G. corallioides*, forming gregarious, black perithecioid fruitbodies on the lower parts of host shoots. The study expands the known distribution of the fungus from a stronghold in northern and northeastern Europe to include the Aleutian Islands in Alaska. One of the host bryophytes, *G. corallioides*, is threatened by deterioration of its high-altitude Arctic-montane habitats, especially along the southern parts of its range, including Britain and Ireland. Our work supports the concept that research utilising herbarium material may provide valuable, unexpected outcomes, with bryophytes as important sources of fungi that have been inadvertently collected in the past.

## Introduction

While the fungal associates of vascular plants are generally well understood, those that interact with bryophytes (mosses, liverworts and hornworts) remain poorly studied by comparison (e.g. compare Ellis and Ellis 1985; with Ellis and Ellis 1988; Davey and Currah 2006). This is partly because bryophytes themselves are a less-studied group (“minority taxa” sensu Smith 2020). They are small and their tissues often relatively simple, hence their associated fungi are usually only able to produce small fruitbodies (Döbbeler 1997). The fruitbody, containing spores, is the primary feature required for the morphological identification of fungi. Most bryophilous ascomycetes, the group of fungi known to most often infect bryophytes, produce tiny (< 250 μm in diameter) fruitbodies. This makes them easy to overlook, especially when hidden within sheltered and specific ‘microniches’ on the host bryophyte plants (see Döbbeler 2002). *Bryochiton microscopicus* Döbbeler & Poelt, for example, is frequently found on the leafy liverwort genus *Gymnomitrion* Corda (Döbbeler 1978) but is hardly recorded or even noted by bryologists and mycologists.

Some bryophilous fungi have gained much attention in recent years and are being studied intensely, especially the so-called bryoparasitic Pezizales (e.g. Janošík et al. 2023), which include the large and conspicuous species *Neottiella rutilans* (Fr.) Dennis and *Octospora humosa* (Fr.) Dennis. These ‘disc fungi’ are parasitic on Polytrichaceae, often in heathland, and seem to be familiar to many field mycologists but are usually overlooked by bryologists. Most of the other groups of fungi remain understudied, especially smaller and less-conspicuous species. These include most perithecioid species, which form their spores in closed, globose to flask-shaped fruitbodies rather than the open discoid apothecia of *Octospora* Hedw. species. While some perithecioid species are conspicuous in the field due to orange or black coloration, many are minute, contrast poorly with their host plants, and cannot be detected easily, sometimes not at all, until material is carefully examined under a stereomicroscope.

Randomly collecting host material to screen under the stereomicroscope is one way to find some of these species, but this is limited to narrow geographical ranges and restrictions imposed on collecting. Collecting eDNA to assess distributions is currently not possible due to limited taxonomic clarity and a lack of molecular data from many species. An additional approach is the utility of bryophyte herbaria, which often house fungi inadvertently collected on and preserved with their host bryophytes. There are several caveats associated with using herbarium material, notably the deterioration of material and loss of useful identification features, but field-based approaches cannot provide biological information with comparable spatial and temporal breadth. Herbaria reduce the need for expensive and time-consuming fieldwork, especially in remote locations. Additionally, with many herbaria holding collections dating back hundreds of years, they provide unique evidence on historical distributions affected by human-driven climate and habitat change since the Industrial Revolution and the more recent Agricultural Revolution (e.g. Lang et al. 2018; Rosche et al. 2022; Buldrini et al. 2023; Crittenden et al. 2023).

In his Ph.D. project, Peter Döbbeler was the first to utilise herbarium material to study perithecioid ascomycetes on bryophytes, highlighting the untapped potential of bryological herbaria for taxonomic and biogeographical studies on bryophilous fungi (Döbbeler 1978). Subsequent studies revealed new and diverse fungi. For example, 21 different species of ascomycetes, including species with highly reduced morphology, were discovered on 51 herbarium specimens of *Dawsonia* R. Br. originally collected in Australasia (Döbbeler 1981). Screening of Finnish herbarium material comprising around 400 vouchers of *Plagiochila asplenioides* (L.) Dumort. and *P. porelloides* (Torr. ex Nees) Lindenb. resulted in 200 new fungal records, with six fungi new for the country (Marsh et al. 2010). The systematic screening of various bryophytes resulted in the first major biogeographical mapping study of 21 bryophilous fungi (Döbbeler and Hertel 2013). These studies demonstrate that herbaria are excellent, largely untapped sources of bryophilous fungi.

Here, we present a case study where we utilised herbarium material to discover new records of the bryophilous ascomycete *Bryobroma gymnomitrii* (Döbbeler) Döbbeler (≡ *Bryomyces gymnomitrii* Döbbeler) on *Gymnomitrion concinnatum* (Lightf.) Corda and *G. corallioides* Nees historically collected in Britain and North America. Several other members of the family Gymnomitriaceae were screened, including other *Gymnomitrion* species as well as multiple *Marsupella* Dumort. species. This is because the systematic placements of some taxa (e.g. *G. alpinum* (Gottsche ex Husn.) Schiffn., syn. *M. alpina* (Gottsche ex Husn.) Bernet have moved between these closely related genera over time (Vilnet et al. 2010; Mamontov et al. 2019). The collectors did not note the presence of the fungal fruitbodies on their liverwort samples on the labels of any of the vouchers we reviewed. The North American records significantly expand the known range of *B. gymnomitrii*. A brief description of the fungus, adapted from the original description by Döbbeler (1978), along with newly generated micrographs, are provided to aid in future identifications of the species. This study highlights the value of biological collections, particularly in terms of their capacity to store additional data not necessarily gathered with intent by the collectors.

## Materials and methods

Herbarium material of *Gymnomitrion* and *Marsupella* (Gymnomitriaceae) from ALA, BBSUK, NMW and OSC was screened under stereomicroscopes. Liverwort fragments of interest were hydrated, and fungal structures were examined under high-power compound light microscopes. Approximately 43 vouchers from OSC were studied by M.H., plus personal collections of M.H. (20) and Professor Bruce McCune (10). All specimens examined were *G. obtusum* (64) except *G. concinnatum* (5), *G. corallioides* (2) and *G. pacificum* (2). A total of 83 vouchers from ALA were studied by W.C.B., most of which were made up of *G. concinnatum* (25) and *G. corallioides* (18), along with smaller numbers of *G. obtusum* (15), *Maruspella apiculata* (syn. *G. apiculatum*; 13), *G. pacificum* (9) and *G. revolutum* (3). Approximately 100 *Gymnomitrion* vouchers and 160 *Marsupella* vouchers (according to the voucher labels) from BBSUK and NMW were studied by G.R.L.G. (in addition to a few personal collections), which included most British and Irish *G. corallioides* vouchers (∼25), *G. concinnatum* from multiple British and Irish locations (∼25 vouchers), *G. obtusum* (∼30 vouchers) and *G. crenulatum* (∼25 vouchers). Most BBSUK *Marsupella* vouchers were also screened due to frequent taxonomic changes between *Gymnomitrion* and *Marsupella* (with the exception of *M. aquatica* and *M. emarginata*): *M. adusta* (now *G. adustum*; 16), *M. alpina* (now *G. alpinum*; 18), *M. boeckii* (5), *M. brevissima* (18), *M. condensata* (7), *M. funckii* (∼30), *M. profunda* (2), *M. sparsifolia* (2), *M. sphacelata* (∼35), *M. sprucei* (∼25) and *M. stableri* (8). See Table 1 for a summary of the above. Bryophyte nomenclature follows Hodgetts et al. (2020). In NMW and BBSUK, fragments from specimens containing fungal structures were placed in labelled sub-packets and stored within the original host bryophyte voucher along with determination slips so the collections were not split. In OSC and ALA, fungus-containing material was returned unaltered to the original bryophyte packet, making note of the voucher codes and the fungi found in them.

**Table 1.** Summary of the total number of vouchers and species of Gymnomitriaceae screened in the present study.

Species	No. of vouchers screened
*Gymnomitrion adustum* Nees	16
*Gymnomitrion alpinum* (Gottsche ex Husn.) Schiffn.	18
*Gymnomitrion brevissimum* (Dumort.) Warnst.	18
*Gymnomitrion concinnatum* (Lightf.) Corda	55
*Gymnomitrion corallioides* Nees	45
*Gymnomitrion crenulatum* Gottsche ex Carrington	25
*Gymnomitrion obtusum* Lindb.	109
*Gymnomitrion pacificum* Grolle	11
*Gymnomitrion revolutum* (Nees) H.Philib.	3
*Marsupella apiculata* Schiffn.	13
*Marsupella boeckii* (Austin) Lindb. ex Kaal	5
*Marsupella condensata* (Angstr. ex C.Hartm.) Lindb.	7
*Marsupella funckii* (F.Weber & D.Mohr) Dumort.	30
*Marsupella profunda* Lindb.	2
*Marsupella sparsifolia* Lindb.	2
*Marsupella sphacelata* (Giesecke ex Lindenb.) Dumort.	35
*Marsupella sprucei* (Limpr.) Bernet	25
*Marsupella stableri* Spruce	8
Total	427

GBIF (https://www.gbif.org/; GBIF Secretariat) was consulted for records of *Bryobroma gymnomitrii* (≡ *Bryomyces gymnomitrii*) outside the original description. We generated the map image on R (R Core Team 2021), using the R packages *maps* (version 3.4.1.1, Brownrigg 2023) and *ggplot2* (version 3.4.0, Wickham 2016). We extracted the world polygons from the *maps* package and plotted the *B. gymnomitrii* occurrence points using *ggplot2* and projection option *ortho*, focusing on the North Pole.

## Results

### Description and identification of *Bryobroma gymnomitrii*

***Bryobroma gymnomitrii*** (Döbbeler) Döbbeler, Ascomycete.org 16(2): 15 (2024).

(Figure 1)

**Figure 1.**
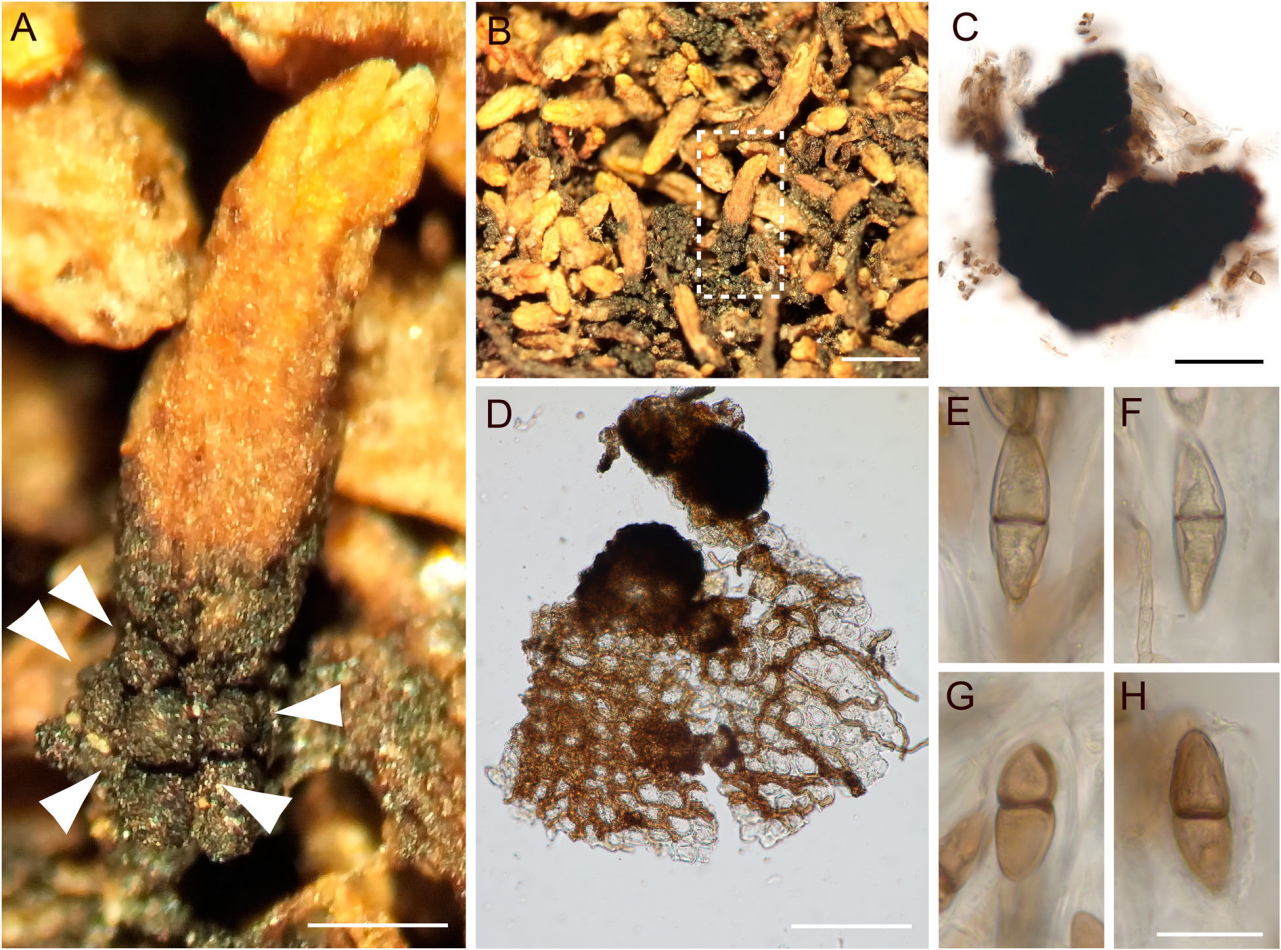
*Bryobroma gymnomitrii* on *Gymnomitrion concinnatum*. (A) Infected host shoot with blackened lower region and shiny, black ascomata (arrowed). (B) Dry herbarium material indicating the shoot magnified in (A). (C) Ruptured ascoma in water with free ascospores in solution. (D) Young ascomata among mycelium on an infected host leaf with dense stroma to the left of the image. Note the thick-walled, brown, large hyphae. (E–H) Ascospores in water. Scale bars: A = 0.25 mm, B = 1 mm, C and D = 100 μm, E–H = 20 μm. A, B, D from *F. E. Milsom*, 1924, Wales (BBSUK C.2001.020.728); C, E–H from *W. B. Schofield* 116349 et al., 2000, Alaska (ALA B35845).

= *Bryomyces gymnomitrii* Döbbeler, Mitt. Bot. Staatsamml., Münch. 14: 241 (1978).

**Description.** Ascomata perithecioid, frequently gregarious and occasionally laterally fused together, globose, black, shiny, glabrous, (100–)150–250 μm in diameter, developing on a blackish stroma. The cells of the outer layer of the peridium roughly isodiametric with thick, dark walls. Paraphysoids formed as scanty fragments or absent. Asci bitunicate, ovoid to ellipsoidal, with a short, narrowed foot, broadly rounded and thickened at the apex, 8-spored, inamyloid, 52–75 μm. Ascospores ellipsoidal to almost fusiform, 1-septate, grey-brown, halves unequal, smooth-walled, not or slightly constricted at the septum, 28–35(–39) × 8–11 μm. Mycelium below the fruitbodies often forming stroma-like tissue comprising overlapping, anastomosing hyphae, one to several cells thick, preferentially growing along the anticlinal cell walls to form a web-like appearance. Hyphal cells dark brown, thick-walled, 3.5–8 μm wide, sometimes growing within host cells but mostly superficial. Haustoria hyaline to brown, pin-shaped, about 1 μm wide, produced on the ventral parts of the hyphae in contact with the host cells, penetrating into the host cell walls, apices occasionally branched. Description adapted from Döbbeler (1978).

**Hosts.** *Gymnomitrion concinnatum* and *G. corallioides*.

**Figure 2.**
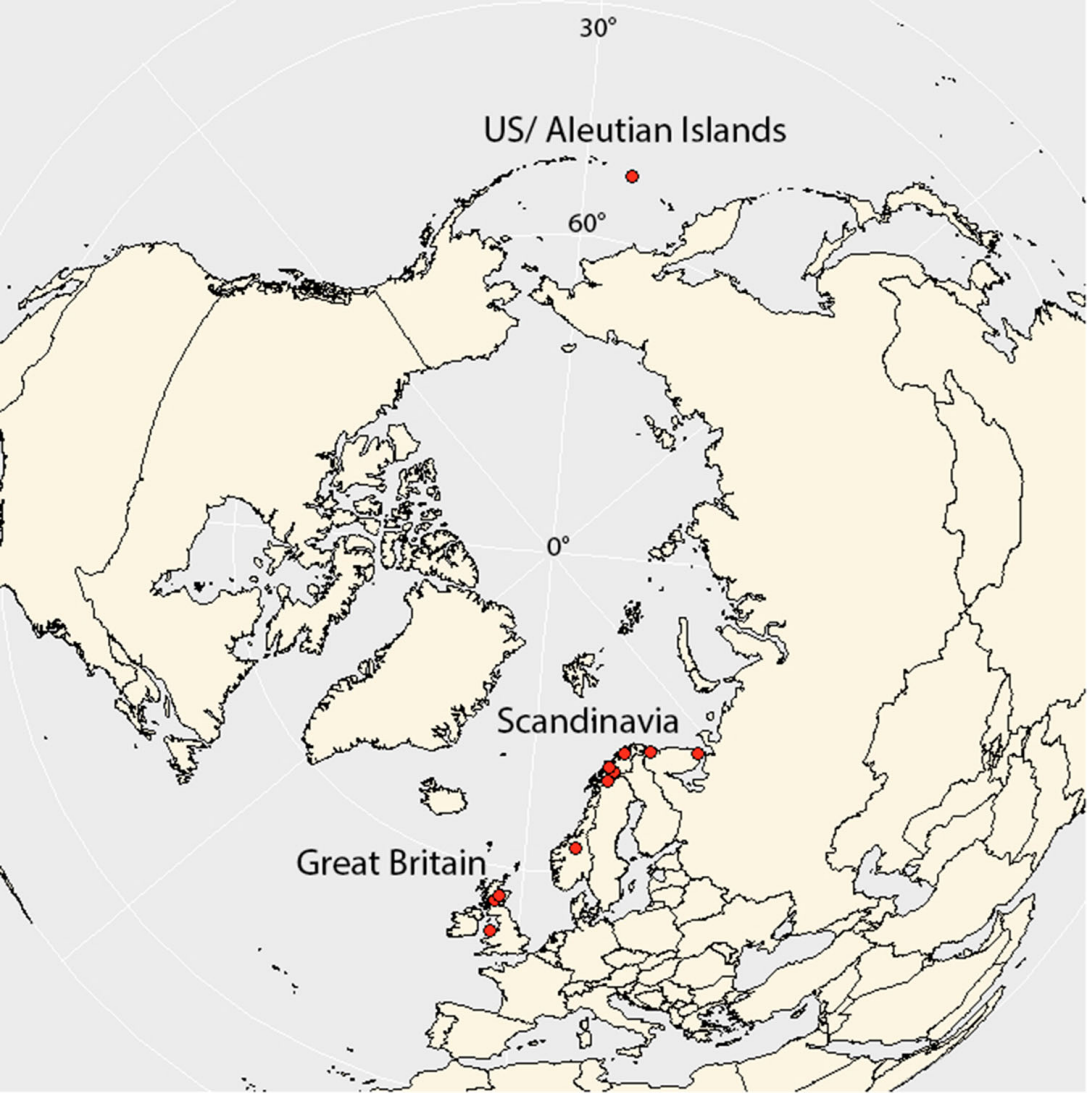
Map showing the distribution of *Bryobroma gymnomitrii* based on GBIF data augmented with the new British and North American records presented here.

The holotype specimen of *Bryobroma gymnomitrii*, deposited in GZU (catalogue number GZU 000312194; isotype in M), was collected in Sweden in August 1972 along with some additional collections (deposited in UPS and ZT) made during the same expedition, all of which were published alongside the type (Döbbeler 1978). No records have been published in a peer-reviewed journal since then, but additional records (5 human observations and 12 herbarium specimens) have been published on GBIF (https://www.gbif.org/; GBIF Secretariat 2023). These include records from Finland, Norway, Sweden and northwestern Russia, some of which appear to have been duplicated (Figure 2; Supplemental material 1).

*Bryobroma gymnomitrii* is distinctive as it turns the lower parts of infected host shoots black due to its often-gregarious ascomata and dense, dark-brown, stromatic mycelium. The ascomata are larger than those of associated species, including the frequently occurring *Bryochiton microscopicus*. The combination of characters, especially mycelium and spore morphology, separates *B. gymnomitrii* from all other known fungi and lichens that associate with *Gymnomitrion*, especially species of *Atla* (lichenised with giant muriform spores) and *Pleostigma jungermannicola* (C.Massal.) Kirschst. (with brown muriform spores). Our specimens correspond with the detailed description of the holotype (Döbbeler 1978).

This pilot study, ahead of a more in-depth review of the fungi on *Gymnomitrion*, serves to illustrate to a bryological audience that bryologists can use bryophyte herbaria to study the relationships between bryophytes and fungi, as well as collect new regional records of understudied species. Even if fungi are not studied in detail, noting the possible presence of fungal structures on the herbarium packet could be extremely useful for future studies in bryo-mycology that screen those specimens.

### New records for North America and the British Isles

**Europe.** Scotland: Mid-Perthshire (v.-c. 88), Ben Lawers, on lower shoots of *Gymnomitrion corallioid*es at margins of patch, 1 August 1908, *H. H. Knight* (NMW C91.33.44) – Ben Lawers, on lower shoots of *G. corallioides*, August 1907, *H. H. Knight* (NMW C91.33.43). – Aberdeenshire (v.-c. 92), Ben Macdhui, on *G. corallioides* with *Bryochiton microscopicus* and *Sclerococcum* sp., July 1884, *G. Stabler* (NMW C.1999.028.3352; NMW C44.66.332). Wales: Caernarvonshire (v.-c. 49), Snowdon, Clogwyn Rocks, on *Gymnomitrion concinnatum*, August 1924, *F. E. Milsom* (BBSUK C.2001.020.728).

**North America.** Alaska: Aleutian Islands, Attu Island, Robinson Ridge, on *Gymnomitrion concinnatum*, outcrop on ridge, 14 August 2000, *W.B. Schofield 115748* and *S.S. Talbot* (ALA B36494). – Henderson River, on *Gymnomitrion concinnatum*, cliff crevices and face, 29 August 2000, *W. B. Schofield 116349*, *S. S. Talbot* and *S. Looman Talbot* (ALA B35845).

## Discussion

The genus *Bryobroma* contains nine species, one of which (*B. microcarpum* (Döbbeler) Döbbeler) has three varieties (Döbbeler 1978; Greiff and Döbbeler 2024). *Bryobroma microcarpum* and *B. velenovskyi* (Bubák) Döbbeler, which grow on mosses, seem to be the most commonly found species. Most of the remaining species grow on liverworts and have rarely or not been recorded since their original descriptions, including *B. hemisphaericum* (Döbbeler) Döbbeler on *Plagiochila porelloides* and the type species, *B. scapaniae* (Döbbeler) Döbbeler on *Scapania undulata* (L.) Dumort. (Döbbeler 1978; Marsh et al. 2010; Hyde et al. 2017). Molecular data from fresh collections, especially of *B. scapaniae*, could aid significantly in understanding the natural systematic position of the genus. Working with the genus is challenging as few collections exist, species delimitations are not straightforward (e.g. *B. microcarpum* agg.), and the quality and quantity of fruitbodies with spores are often deficient (Döbbeler 1978; Döbbeler 2003).

While the presence of *Bryobroma gymnomitrii* in Britain and Ireland is not unusual given the known European distribution of the fungus so far, the Alaskan records are highly significant and suggest that *B. gymnomitrii* has a much wider distribution, coinciding with that of its known hosts, *Gymnomitrion concinnatum* and *G. corallioides* (see Figure 2). That said, however, *B. gymnomitrii* appears to be absent from the Alps, despite concentrated recording efforts (Döbbeler 1978). This may be due to the relatively recent geological emergence of the Alps, coupled with a poor dispersal ability of the fungus. *Bryobroma gymnomitrii* was not reported in a recent synoptic study detailing the bryophilous ascomycetes recorded in North America, nor indeed were any other species of *Bryobroma* (Döbbeler et al. 2023). However, the fungus can be easy to dismiss or overlook unless it is specifically searched for with prior knowledge. For example, mycelium matching *B. gymnomitrii* is clearly visible on the leaves of two collections of *G. corallioides* photographed from eastern Russia in Bakalin (2016, p.59), but confirmation of these records would require detailed examination of the vouchers in question.

Despite 18 different species of *Gymnomitrion* and *Marsupella* having been screened, *Bryobroma gymnomitrii* was observed on only *G. concinnatum* and *G. corallioides*. This could indicate some measure of host or ecological specificity. *Gymnomitrion concinnatum* is described as an Arctic-montane species that forms yellowish-green to greyish patches on siliceous rocks and gravelly or mineral soils in exposed to sheltered montane habitats (Blockeel et al. 2014). This source also describes a decline in *G. concinnatum* in Britain and Ireland along the southern part of its range, which includes Yr Wyddfa (Snowdon), the location of the 1923 record in Wales. On the other hand, *G. concinnatum* is the most commonly reported *Gymnomitrion* species in the Aleutian Islands, Alaska (Davison 1993), including Attu Island, where *B. gymnomitrii* was found to co-occur with it (Talbot et al. 2018). The other host, *G. corallioides*, is a circumpolar, Arctic-montane species with an interestingly broad ecological niche, growing on base-rich and acidic rocks (Blockeel et al. 2014). The species is rarely recorded in Britain and Ireland, and is presumed to be extinct in Wales, having last been recorded in Eryri (Snowdonia) in 1912. It may, however, be mistaken for the more common *G. obtusum* in the field. Like *G. concinnatum*, it is common in appropriate habitats in the Aleutians and is the co-dominant species with the frequently associated hepatic *Anthelia juratzkana* (Limpr.) Trevis. on recent pyroclastic flows from Aniakchak Volcano on the Alaska Peninsula (Boggs et al. 2019). *Gymnomitrion corallioides* has a generally high altitudinal niche so is more threatened by climate and habitat change than many of its relatives as climatically suitable habitats reduce in an ‘escalator to extinction’ effect (Britton et al. 2018; Urban 2018; Alatalo et al. 2020; Weldon et al. 2022).

Both host species of *Bryobroma gymnomitrii* frequently grow with other Gymnomitriaceae members, so it is unclear why they are favoured over other related species. While ecological and biochemical factors may explain the host selection, we cannot eliminate the possibility that other hosts have not been observed due to biases, not only in the composition of the collections examined but also in relation to the behaviour and constraints of the authors. For example, *B. gymnomitrii* specifically grows on older parts of host shoots, a niche also occupied by *Pleostigma jungermannicola* and lichenised *Atla* species, which seem to be much more common and look very similar to *B. gymnomitrii*, especially in the dry state when specimens are primarily screened. Screening dry material means that many of the older parts of the host shoots will be buried within the mats, so fungi in these regions will often not be visible and can be overlooked.

There are several general challenges and caveats associated with the use of herbarium material that may have contributed to the relatively low number of records generated. First, the condition of different bryophytes and fungi deteriorate to different degrees during processing (e.g. cold treatments for pests) and subsequent storage in the herbarium, where some useful taxonomic characters are lost and colour contrasts visible in fresh material disappear. These characters include the loss of oil bodies in liverworts (Paton 1999). The identification of bryophilous fungi often requires destructive analysis, where tiny portions of the original specimen are destroyed in the process of dissecting and mounting the fungi. This involves minimal damage to host tissues, comparable to traditional bryological studies where a small number of leaves may be removed for microscopy, and the problem can be mitigated by preserving the removed material in microscope slides. Rehydration of specimens is considered a threat to other applications, such as DNA sequencing, so primary screening of specimens (under a dissecting microscope) is often requested to be carried out in the dry state, with small fragments removed for hydration and microscopy. While this may help to preserve the specimens, it severely limits the ability to detect fungal structures, which can be buried within host mats and difficult or impossible to spot unless specimens are hydrated (e.g. Döbbeler 2002). Infections are often patchy in host populations, so rehydrating small fragments may not capture the full range of fungi associated with a particular specimen. Finally, the collections of different herbaria have different compositions due to biases in the preferences and intentions of the collectors and curators over time. For example, modern bryologists in Britain and Ireland usually collect small amounts of material, down to single shoots, for specimens requiring microscopic confirmation. Generally, any obviously discoloured patches or parts of patches that may harbour fungi are not collected, in favour of typical material (Döbbeler 1997). Because fungal infections are patchy and often not prolific on healthy or vigorous bryophyte material, smaller modern collections are less likely to harbour good material of fungi compared with (usually older) collections comprising larger host mats with more variation in the individual shoots present.

The present study provides proof of the concept that interesting, under-recorded fungi are present within bryophyte herbarium specimens, and often overlooked or dismissed by the collectors. Herbarium material of specific bryophytes can be targeted to improve our understanding of the fungal associates of those species across time and space, as well as to inform on areas to target for future fieldwork. This is particularly pertinent as climate change continues to threaten arctic-alpine specialists, including many *Gymnomitrion* and *Marsupella* species. Bryologists are particularly well equipped to contribute towards this field, already possessing skills in identifying the host plants, a vital step towards identifying any fungi that may be present on them.

## Supplementary Material

JBR 2059 Supplemental material 1.pdf

## Supplemental material

**Supplemental material 1.** *Bryobroma gymnomitrii* mapping datapoints and sources of those data.
