# Supplementary material for: Utilising bryophyte herbarium material as a source of fungal novelty: a case study presenting new records of Bryobroma gymnomitrii (Döbbeler) Döbbeler on Gymnomitrion Corda in Britain and North America
Source: J Bryol. 2024 Jul 29;46(3):215–22. doi: 10.1080/03736687.2024.2375407 (PMC11614047; doi:10.1080/03736687.2024.2375407)
Supplement: JBR 2059 Supplemental material 1.pdf [file YJBR_A_2375407_SM5211.pdf]

SUPPLEMENTARY TABLE S1

GBIF ID	Country	Locality data	Lat	Long	Date (YYYY/MM/DD)	Institution code	Identifier	Co-ordinate source	Reference
**4023883422**	Russia				1883-06-01	H	Anne Lesonen	Not provided	GBIF; https://doi.org/10.15468/dl.tbmzcx
**4023883352**	Russia				1883-06-01	H	Not provided (Anne Lesonen?)	Not provided	GBIF; https://doi.org/10.15468/dl.tbmzcx
**4023881924**	Russia	Liinahamari.	69.64027	31.342842	1933-07-06	H	Not provided (Anne Lesonen?)	Estimated from locality	GBIF; https://doi.org/10.15468/dl.tbmzcx
**4023881923**	Russia	Liinahamari.	69.64027	31.342842	1933-07-06	H	Anne Lesonen	Estimated from locality	GBIF; https://doi.org/10.15468/dl.tbmzcx
**4023873376**	Finland	Lappi	68.9966	20.851941	1935-07-09	H	Not provided (Anne Lesonen?)	GBIF	GBIF; https://doi.org/10.15468/dl.tbmzcx
**4023873353**	Finland	Lappi	68.9966	20.851941	1935-07-09	H	Anne Lesonen	GBIF	GBIF; https://doi.org/10.15468/dl.tbmzcx
**4023873351**	Finland	Lappi	68.9966	20.851941	1935-07-09	H	Not provided (Anne Lesonen?)	GBIF	GBIF; https://doi.org/10.15468/dl.tbmzcx
**4023872912**	Finland	Lappi	69.12597	20.57579	1962-07-11	H	Anne Lesonen	GBIF	GBIF; https://doi.org/10.15468/dl.tbmzcx
**4023872890**	Finland	Lappi	69.04604	20.850864	1966-08-16	H	Anne Lesonen	GBIF	GBIF; https://doi.org/10.15468/dl.tbmzcx
**4023871588**	Finland				1883-08-01	H	Anne Lesonen	Not provided	GBIF; https://doi.org/10.15468/dl.tbmzcx
**4023871585**	Finland				1883-08-01	H	Not provided (Anne Lesonen?)	Not provided	GBIF; https://doi.org/10.15468/dl.tbmzcx
**4023869490**	Russia	Ponoi	67.078	41.119	1863-06-01	H	B.J. Coppins	GBIF	GBIF; https://doi.org/10.15468/dl.tbmzcx
**351744924**	Sweden	Nordhänge des Njulla (Nolja) südlich oberghalb der bahnstation, [illegible]			1972-08-14	UPS	Not provided	Estimated from locality	GBIF; https://doi.org/10.15468/dl.tbmzcx
**335384725**	Russia	Ponoi	67.078	41.119	1863	H	Not provided (B.J. Coppins?)	GBIF	GBIF; https://doi.org/10.15468/dl.tbmzcx
**2867324695**	Norway	Steisandmoen	69.7	20.1	1916-07-17	O	Marie Louise Davey	GBIF	GBIF; https://doi.org/10.15468/dl.tbmzcx
**2867324524**	Norway	Holmen	70.47	25.06	1894-07-26		Marie Louise Davey	GBIF	GBIF; https://doi.org/10.15468/dl.tbmzcx
**2867324377**	Norway	Kongsvold	62.3	9.8	1907-07-17		Marie Louise Davey	GBIF	GBIF; https://doi.org/10.15468/dl.tbmzcx
**1928155871**	Sweden	Torne Lappmark. Jukkasjärvi sn.: Umgebung von Abisko, NW- [illegible]	68.4	18.68	1980-08-02	NY	P. Döbbeler	GBIF	GBIF; https://doi.org/10.15468/dl.tbmzcx
**1638380317**	Sweden	Torne Lappmark: nahe dem See Kärkevaggepadajaure, 4 km S des [illegible]	68.4	18.68	1972-08-23	GZU	P. Döbbeler	Estimated from locality	GBIF; https://doi.org/10.15468/dl.tbmzcx
**NA**	Wales	Snowdon	53.06853	-4.075375	1924-08	BBSUK	G.R.L. Greiff	Estimated from locality	This study
**NA**	Scotland	Ben Lawers	56.5452	-4.221747	1908-08-01; 1907-08	NMW	G.R.L. Greiff	Estimated from locality	This study
**NA**	Scotland	Ben Macdhui	57.07041	-3.669607	1884-07	NMW	G.R.L. Greiff	Estimated from locality	This study
**NA**	Alaska	Attu Island: Henderson River	52.89037	173.15573	2000-08-14	ALA	W.C.Bleecker	Estimated from locality	This study
**NA**	Alaska	Attu Island: Robinson Ridge	52.89037	173.15573	2000-08-29	ALA	W.C.Bleecker	Estimated from locality	This study
